# Endorsement of scientific norms among non-scientists: The role of science news consumption, political ideology, and science field

**DOI:** 10.1177/09636625251315882

**Published:** 2025-02-28

**Authors:** Markus Schug, Helena Bilandzic, Susanne Kinnebrock

**Affiliations:** University of Augsburg, Germany

**Keywords:** controversial science, political ideology, science news, scientific norms

## Abstract

Public discussions of controversial science fields like COVID-19 or climate science increasingly address inner-scientific structures and the norms guiding the scientific system—aspects that are normally discussed within the scientific community. However, not much is known about the endorsement of scientific norms by non-scientists and how those endorsements differ between controversial und uncontroversial science fields. We conducted a cross-sectional national survey in Germany (*N* = 1007) to capture the public endorsement of scientific norms and explored the role of the science field, political ideology, and science news consumption. Results suggest that the endorsement of scientific norms is significantly higher in controversial fields than in less controversial fields. More left-leaning political ideology is connected to higher levels of norm endorsement; science news consumption is partly associated with lower scientific norm endorsement. We discuss our findings regarding their implications for the public’s image and understanding of controversial science fields.

Scientists follow professional norms in their daily work—they strive to make their methods and results public and transparent to enable critical scrutiny and replication, minimize and disclose conflicts of interest, subject their work to the scrutiny of peers and present original and innovative work. While these standards are constantly evolving (as the open science movement powerfully demonstrates), the basic idea of “scientific norms” by Robert K. Merton (1973) still resonates with many academic researchers and is still visible in practices that characterize and define academic research. Norms are defined as implicit or explicit rules that indicate which behaviors are appropriate in a particular social field and which are not (Lapinski and Rimal, 2005; Young, 2008). Relating to science, norms are prescriptions governing the work of scientists, assuring the quality of the results and acceptance by peers (Merton, 1973; Ziman, 2000).

Normally, scientific norms are negotiated within the scientific community. In some politicized and controversial domains of science however, these norms become the subject of public debate as well. Such controversial fields are sometimes called “post-normal” science—science domains in which “facts are uncertain, values in dispute, stakes high and decisions urgent” (Funtowicz and Ravetz, 1993: 744; Roger, 2017; Turnpenny et al., 2010). Examples for such contested domains under close public surveillance are climate change (Saloranta, 2001) and the COVID-19 pandemic (Waltner-Toews et al., 2020). Quality assessment and thus following scientific norms becomes crucial in these domains as policy decisions and ultimately lives are at stake—not just in the traditional sense of an internal monitoring and debate about quality, but also in the sense of involving the public who are directly affected by the research (Funtowicz and Ravetz, 1991; Roger, 2017).

When scientific norms become the subject of public debate, it is notable that controversial fields of science are more connected to norm transgressions rather than norm compliance. Norm compliance is the regular case in social interactions and evolves in a “noiseless,” unspectacular way. It is the norm transgressions that produce irritations and frictions (Jacobsson and Löfmarck, 2008) and ultimately make a case newsworthy, containing conflict, personalization, and harm—as classic news factors would predict (Boukes et al., 2022).

A prominent example of a widely publicized but ultimately inaccurate report of an alleged transgression of scientific norms is the so-called “Climategate” scandal: Emails were hacked from a British university server and leaked on the Internet. Climate skeptics spread the rumor that the emails showed a climate conspiracy staged by climate scientists who supposedly manipulated data and deceived the public. If these allegations were true, they would violate the Mertonian norm of disinterestedness (having no other goals than scientific progress). Even though these allegations were refuted by several investigations, the rumor turned out to be hard to eliminate from public memory (Maibach et al., 2012). Another example from the COVID-19 pandemic is the German case of media debates about the “Heinsberg study” by Dr. Hendrik Streeck or the “Charité Viral Load in Children Study” by Dr. Christian Drosten, both leading German virologists during the COVID-19 pandemic. In both cases, the scientists were attacked by yellow press news coverage using open peer reviews as critical grounds for dismissal of the results and questioning the moral integrity of researchers (Breznau et al., 2020; Piatov, 2020).

Doubting that scientists follow professional norms is a rhetorical strategy of anti-science discourse to cast doubt on the legitimacy of science’s voice in societies and has led to the presence of the corrupted scientist archetype in public discourse (Cloud, 2020). Such discussions discredit the scientific consensus regarding important science topics and consolidated scientific practices of producing reliable knowledge (Brüggemann et al., 2020; Slater et al., 2024). The more a science field is involved in public debate and policy making, the greater the public scrutiny of the actors and their studies, and the greater the standards (in the form of scientific norms) that are attached to them. We consider scientific norms and their support by broader publics as important cues for the public understanding of science.

In this paper, we argue that public endorsement of scientific norms is not equal across all fields of science but depends on the amount of controversy attached to the field, as well as on personal and mediated experiences with science and political ideology. We investigate these factors in a cross-sectional survey (*N* = 1007).

## 1. Scientific norms

According to Merton (1973), the scientific norms *communism* (often referred to as *communalism*, see Ziman, 2000), *universalism, disinterestedness*, and *organized skepticism* together form the “ethos of science,” encompassing basic value orientations that scientists commonly share, even across disciplinary boundaries. Ziman (2000) later added *originality* to this list. Merton (1973) understands these norms as “institutional imperatives” (p. 270) guiding the scientific enterprise.

*Communalism* concerns the availability of scientific knowledge (Merton, 1973). Researchers should be transparent and openly share research, data, and materials that might be relevant to other, also rivaling, scientists (Bray and Von Storch, 2017). Communalism requires that scientific research become a “common property”—primarily for the scientific community, but also for society as a whole (Lewandowsky and Oberauer, 2021). The trend toward Open Access in science, making research available to the public, is based on this demand (Dickel, 2017).

*Universalism* is the independence of research results from demographic criteria of researchers (Merton, 1973). Basically, everyone should be able to contribute to the progress of scientific knowledge without being hindered by divisive barriers (Lewandowsky and Oberauer, 2021: 2). Scientists are required to evaluate scientific findings solely on their merits and accepted field standards, regardless of the personal characteristics of the person making the claim (Anderson et al., 2010).

*Disinterestedness* requires research to be independent from personal advantages (i.e. financial gain, social prestige, scientific advantage; Merton, 1973). As an “individual virtue” (Cloud, 2020: 816–823), disinterestedness addresses the need for explicit and appropriate motivations such as a general “desire for knowledge and discovery” (Anderson et al., 2010: 7) when scientists conduct research. In the context of public perceptions of scientists, it is also a key requirement for avoiding destabilizations of scientific authority (Johnson and Dieckmann, 2020).

*Organized skepticism* embeds systematic doubt in science, whereby no scientific claim should be immune from being questioned (Merton, 1973). Scientists should internalize that any scientific “truth” is provisional, contestable, and replaceable by new knowledge (Dickel, 2017: 57). When scientists conduct their research and draw conclusions, they should consider all available findings and theories, “even those that challenge or contradict their own work” (Anderson et al., 2010: 7).

*Originality*, as added by Ziman (2000), “requires scientists to produce *new* knowledge—that is, communally acceptable information that was not previously known. To do this, they engage in *research*” (Ziman, 2000: 182, emphasis i. o.). Ziman (2000) highlights the gaps in scientific knowledge that leave both societal and scientific questions and problems unresolved. The constant pursuit of new answers, new research, and the filling of research gaps is inscribed in the societal function of researchers (Ziman, 2000).

There has already been research on *scientists’* endorsement of and compliance with these norms (e.g. Anderson, 2000; Anderson et al., 2010; Bray and Von Storch, 2017). Results show partial support and partial deviation from the norms. For instance, in the context of climate scientists, Bray and Von Storch (2017: 1351) found that even though the Mertonian norms remained “overall guiding principles,” they are not “fully endorsed or present” in the daily conduct of researchers. Bray and Von Storch (2017) also emphasize the presence of counter-norms, calling researchers for “potentially contradictory attitudes and behaviors” in their actual daily habits (Mitroff, 1974: 579). Critics of Merton’s concept emphasize that the existence of counter-norms such as “secrecy” or “self-interestedness” (Mitroff, 1974; Mulkay, 1976) demonstrates that norms are too idealized and alienated from the real social structures of the scientific system. While the Mertonian norms were developed some 80 years ago and thus need to be contextualized in the time and practice of that time (Hosseini et al., 2024), the ideas underlying the norms are still compelling for academic research today, both as descriptions of actual practice (such as peer review, conflict of interest disclosures, publication, and transparency) and as ideals guiding scientific inquiry. Within the field of scientific norms, Merton’s analysis remains the “perhaps most well-known and influential” framework (Lewandowsky and Oberauer, 2021: 2).

## 2. Predictors for the endorsement of scientific norms

While several studies have examined scientists’ endorsement of scientific norms, few studies have examined the *public’s* perceptions of scientific norms. One of the few studies was conducted by Johnson and Dieckmann (2020), focusing on audience perspectives on scientists’ motivations, touching the disinterestedness norm. However, they call for research putting audience studies on scientists’ motivations “into the larger picture,” emphasizing the need to integrate normative perceptions more generally into research on the public understanding of science (Johnson and Dieckmann, 2020: 17).

We assume that especially three factors influence the public endorsement of scientific norms. First, most people are confronted with scientific norms and form their opinion on the endorsement of scientific norms based on own (personal) or representative (mediated) experiences with science (e.g. Hmielowski et al., 2014; Wintterlin et al., 2022). Second, the science field being referenced (controversial or uncontroversial) may affect people’s evaluations of scientific norms as the public may hold controversial areas to a higher standard compared to less controversial areas (e.g. Pechar et al., 2018). Third, conservative or liberal worldviews are considered crucial for expectations toward science, so the endorsement of scientific norms may depend on political ideology (e.g. McCright et al., 2013). We will elaborate on these three factors in more detail below.

### Personal and mediated experiences with science

To date, there are no data on how personal experiences with science or the consumption of science news related to the endorsement of scientific norms. However, personal and mediated experiences with science have been investigated in relation to other science-related constructs. For example, personal (past) experiences with science count as important foundations for trustworthiness perceptions (Wintterlin et al., 2022). Hmielowski et al. (2014) found that media use on science topics are related to trust in scientists and global warming beliefs. In a study of 49 countries, Mede (2022) found that in some countries (e.g. Thailand, Nigeria), more use of legacy media is related to more favorable science attitudes, while in other countries, there were no such relationships (e.g. Germany), or, in yet other countries, more use of legacy media was related to more anti-science attitudes (e.g. Turkey, Bangladesh). Certainly, the correlation direction depends on the media content published in each country, and there is often no consistent overall picture in media coverage of science. In our study, we will extend these findings to scientific norms: RQ1: How are personal experiences with science (RQ1a) and science news consumption (RQ1b) related to the endorsement of scientific norms in controversial and uncontroversial fields of science?

### Controversial and uncontroversial fields of science

We hypothesize that norm endorsement strongly depends on the science field being considered. While there are no studies comparing norm endorsement across science fields, we can extrapolate from other science-related assessments. For example, findings from sociology and criminology are perceived as less plausible than findings from neuroscience and physiology, but more plausible than genetics (Sheremet and Deviatko, 2022). In addition, trust in science also depends on the issue at hand; for example, climate science is more trusted than science about genetically modified foods (Pechar et al., 2018). We propose that support for scientific norms varies depending on how visible and publicized, or controversial, the area of research is. Highly visible, publicized, and controversial fields are more intensively covered in the media through news factors catering to media logics (harm, conflict, personalization, etc.) and are thus more salient in the minds of the audience.

Moreover, because such fields more often directly affect people’s lives and freedom (as in the case of the COVID-19 pandemic and climate change), and their impact on daily life entails high costs for individuals, we expect that the public will hold these areas to a higher standard than areas that are less controversial and consequential (H1).

### Political ideology

Few studies have examined public views of scientific norms. One of them has investigated the role of political worldviews: Lewandowsky and Oberauer (2021) found that people with conservative worldviews were less likely to endorse scientific norms. For controversial science, we know that political ideology plays an important role, for example, more conservative political views are associated with less belief in climate change (e.g. Bolin and Hamilton, 2018) and in COVID-19 vaccines and vaccine policies (Kossowska et al., 2021). Conservatives are also less trusting and less supportive of ‘impact science’—science that points to environmental and health consequences of economic production (McCright et al., 2013), which includes controversial areas such as climate change and pandemics. Supporting this finding, Hamilton et al. (2015) found that Democrats were more likely to trust scientists for information about vaccines and climate change. In a longitudinal study, Gauchat (2012) found that trust in science declined among US conservatives from 1974 to 2010, while it remained stable in other groups. Similarly, political ideology could predict the polarization of attitudes toward nuclear power over an 11-year period (Drummond and Fischhoff, 2020).

Building on this line of research, we hypothesize that a more left-leaning political ideology will be associated with stronger support for scientific norms (H2).

Finally, we assume that especially the role of ideology and science news consumption for endorsement of scientific norms may depend on the science field. As no clear predictions can be derived from existing theory or empirical findings, we add two research questions:

Does the relationship between ideology and endorsement of scientific norms depend on the science field (RQ2)?

Does the relationship between science news consumption and endorsement of scientific norms depend on the science field (RQ3)?

## 3. Methods

To address the hypotheses and the research questions, a cross-sectional national survey in Germany was conducted.^1^ We drew quota samples with age (three equal groups: 18–35, 36–59, and 60+ years), sex (two equal groups: male, female), and education (two equal groups: no German degree for higher education, German degree for higher education) serving as quotas. The sample (*n* = 1148) was secured by the German online access panel company Respondi in March 2023. The questionnaire was developed in German. To ensure the accuracy of translations of scales and items derived from non-German research, two researchers fluent in both languages separately checked the match of the German translations. During data cleaning, we eliminated cases of negligent or inattentive responses, based on two attention checks, obviously systematic answer behavior (i.e. “straight-lining”), and implausible response times. The final sample included 1007 respondents fitting the desired quotas (age: *M* = 47.00, *SD* = 18.66; sex: 51.24% female; education: 50.8% with a German degree for higher education). Detailed descriptive statistics and zero-order correlations of all relevant variables are listed in Table 1.

**Table 1.** Descriptive statistics and zero-order correlations of the variables.

		M/%	SD	Min./max. scale	1	2	3	4	5	6	7	8	9	10	11	12
1.	Age	47.00	18.66	18–83	—											
2.	Sex (female)	.51	—	—	−.04	—										
3.	Education (higher)	.51	—	—	.04	.01	—									
4.	Interest in science	4.90	1.54	1–7	.05	−.21***	.28***	—								
5.	Experience with science	.25	—	—	.01	−.06	.26***	.25***	—							
6.	Political ideology	4.88	1.57	1–10	−.03	−.04	−.09**	−.06	−.09**	—						
7.	Science news consumption	2.44	1.26	1–8	−.31***	−.15***	.11***	.30***	.25***	.05	—					
8.	Scientific norms overall	5.33	.85	1–7	.33***	.04	.21***	.35***	.07*	−.16***	−.23***	—				
9.	Communalism	5.35	1.09	1–7	.25***	−.00	.13***	.28***	.05	−.11***	−.12***	.81***	—			
10.	Disinterestedness	5.23	1.01	1–7	.33***	.04	.24***	.30***	.07*	−.16***	−.27***	.86***	.53***	—		
11.	Organized skepticism	5.43	.98	1–7	.31***	.04	.22***	.30***	.08**	−.18***	−.25***	.91***	.61***	.77***	—	
12.	Originality	5.28	1.11	1–7	.13***	.06	.01	.24***	−.03	−.02	−.03	.61***	.50***	.36***	.42***	—

*N* = 1007. Sex (0 =male, 1 = female). Education (0 = lower, 1 = higher). Experience with science (0 = no experience, 1 = experience). Political ideology (1 = left-leaning, 10 = right-leaning).

*** p < .001; **p < .01; *p < .05.

The questionnaire contained an experimental factor—the potential for controversy of a specific science field. After answering questions regarding sociodemographic characteristics, interest in science, and science news consumption, participants were randomly assigned to one of five versions of the questionnaire: From this point on, each group received a different version of the questionnaire with the same items, but using the scientific fields as reference points in the items or the instructions of the questions (controversial fields: *virology* with focus on COVID-19, *n* = 206; *climate sciences, n* = 205; uncontroversial fields: *astrophysics, n* = 192; *science of history, n* = 195). The fifth group received a questionnaire referencing science in general, without specifying a field, and acted as base line (*n* = 209). For example, the item “Scientists should openly share new findings with others” was used in the science in general condition, while the same item read “*Virologists researching COVID-19* should openly share new findings with others*”* in the virology version and preceded by “*Climate scientists*. . .,” “*Astrophysicists*. . .,” “*Historians*. . .” in the other versions. At the questionnaire end, participants received uniform questions on their experience with science and political ideology.

### Measures

The measure for scientific norms was partly adapted from previous studies (Anderson, 2000; Bray and Von Storch, 2017; Lewandowsky and Oberauer, 2021) and translated into German and partly developed from the theoretical literature on scientific norms (Merton, 1973; Ziman, 2000). We created five subscales for each of the scientific norms, to be used separately and as a composite scale, to be answered on 7-point Likert-type scales from 1 = “do not agree at all” to 7 = “totally agree.” For all items, we manipulated the science field according to the experimental condition. For (1) communalism, we developed six items (for a complete overview of all measures, see Supplemental Material Appendix I “Scales and items”). We eliminated one item because of a lack of consistency with the construct and built a communalism scale of the remaining items (α = .78, *M* = 5.35, *SD* = 1.09). For (2) universalism, we adapted four items. The reliability was low (α = .24) and could not be increased higher than α = .47 by eliminating one or two items. We therefore excluded the norm “universalism” from further analysis. Regarding (3) disinterestedness, we adapted six items. Reliability was good (α = .70). The items were combined into one disinterestedness scale (*M* = 5.23, *SD* = 1.01). For (4) organized skepticism, we adapted seven items. Reliability was good (α = .78). The items were combined into one organized skepticism scale (*M* = 5.43, *SD* = .98). Regarding (5) originality, we developed four items based on the theoretical descriptions by Ziman (2000). We eliminated one item because of a lack of consistency with the construct and built an originality scale of the remaining items (α = .75, *M* = 5.28, *SD* = 1.11). Finally, we combined all 21 remaining items of the four reliable subscales into one scientific norms overall scale (α = .89, *M* = 5.33, *SD* = .85).

Interest in science was measured with a scale by Marschall et al. (2011) and Retzbach and Maier (2015). We used four items on respondents’ interest toward science and scientific issues. The reliabilities were high (α = .95). The four items were combined into a scale (*M* = 4.90, *SD* = 1.54).

Experience with science was measured by two items, adapted from Wissenschaft im Dialog (2021), asking respondents (1) if they know a scientists personally (0 = “no,” 1 = “yes”) and (2) if the respondents themselves work in science or have worked in science (0 = “no,” 1 = “I worked in science in the past,” 2 = “I work in science”). We combined the items into one score, assigning 0 if both items were answered with “no,” and 1 if one or both answers were greater than 0 (0 = “no experience,” 1 = “experience,” *M* = .25).

Political ideology was measured with three items on participants’ ideology toward (1) political, (2) economic, and (3) social issues (Huber et al., 2019). Respondents placed themselves on a scale from 1 = strong liberal (left-leaning) to 10 = strong conservative (right-leaning). The reliabilities were high (α = .88). The three items were combined into a scale (*M* = 4.88, *SD* = 1.57).

Science news consumption was measured with 10 items, each representing a different medium, asking respondents how often in the past 2 weeks they came across information or content from science in various media. We captured the answers on an 8-point scale from 1 = “not at all” to 8 = “many times per day” (newspaper: *M* = 2.77, *SD* = 1.66; magazines: *M* = 2.42, *SD* = 1.67; radio: *M* = 2.48, *SD* = 1.71; TV: *M* = 3.16, *SD* = 1.77; social media: *M* = 2.87, *SD* = 2.17; Wikipedia: *M* = 2.21, *SD* = 1.59; Internet sites on scientific topics: *M* = 2.54, *SD* = 1.69; podcasts: *M* = 1.76, *SD* = 1.43; messaging services: *M* = 2.11, *SD* = 1.97; scientific publications: *M* = 2.09, *SD* = 1.47). The 10 items were also combined into a science news consumption scale (*M* = 2.44, *SD* = 1.26, α = .90), representing the amount of science news that people regularly encounter in the 10 media we measured, independently from the exact source.

## 4. Results

### Preliminary analyses

We first tested the main effects of the experimental conditions on the endorsement of scientific norms in the five experimental conditions with five analyses of covariance (ANCOVAs; controlling for age, sex, education, interest in science, experience with science, political ideology, and science news consumption across media types). We found that the experimental conditions had a significant effect on the endorsement of scientific norms overall (*F* (4, 1001) = 44.21, *p* < .001, *η_p_*^2^ = .33), and more specifically, on the endorsement of communalism (*F* (4, 1001) = 20.88, *p* < .001, *η_p_*^2^ = .19), disinterestedness (*F* (4, 1001) = 41.72, *p* < .001, *η_p_*^2^ = .32), organized skepticism (*F* (4, 1001) = 39.08, *p* < .001, *η_p_*^2^ = .30), and originality (*F* (4, 1001) = 11.37, *p* < .001, *η_p_*^2^ = .11).

Sidak post hoc tests (see Table 2) then found that for scientific norms overall, endorsement values for virology (Estimated Marginal Mean (EMM) = 5.46, *SE* = .05) were significantly higher compared with astrophysics (EMM = 5.21, *SE* = .05, *p* = .00) and science of history (EMM = 5.25, *SE* = .05, *p* = .020). In addition, values for climate science (EMM = 5.42, *SE* = .05) were significantly higher compared with astrophysics (EMM = 5.21, *SE* = .05, *p* = .03). The other group comparisons regarding scientific norms overall were not significant. Sidak post hoc tests for the impact of the experimental conditions on the endorsement of communalism found that values for virology (EMM = 5.52, *SE* = .07) were significantly higher compared with astrophysics (EMM = 5.17, *SE* = .07, *p* = .00) and science in general (EMM = 5.18, *SE* = .07, *p* = .01). In addition, values for climate science (EMM = 5.58, *SE* = .07) were significantly higher compared with astrophysics (EMM = 5.17, *SE* = .07, *p* < .001), science of history (EMM = 5.28, *SE* = .07, *p* = .03), and science in general (EMM = 5.18, *SE* = .07, *p* < .001). The other group comparisons regarding the endorsement of communalism were not significant. Regarding the endorsement of disinterestedness, Sidak post hoc tests for the impact of the experimental conditions found no significant differences between the five experimental conditions. Sidak post hoc tests regarding the impact of the experimental conditions on the endorsement of organized skepticism only found differences concerning the significantly higher values for virology (EMM = 5.56, *SE* = .06) compared with astrophysics (EMM = 5.32, *SE* = .06, *p* = .04). Finally, Sidak post hoc tests for the impact of the experimental conditions on the endorsement of originality found that values for virology (EMM = 5.50, *SE* = .07) were significantly higher compared with astrophysics (EMM = 5.09, *SE* = .08, *p* = .01) and science of history (EMM = 5.11, *SE* = .08, *p* = .01). The other group comparisons regarding the endorsement of disinterestedness were not significant.

**Table 2.** Estimated marginal means (EMM) and standard errors (SE) regarding the endorsement of scientific norms overall, communalism, disinterestedness, organized skepticism, and originality across five experimental conditions (controlling for age, sex, education, interest in science, experience with science, political ideology, science news consumption).

	Virology		Climate science		Astrophysics		Science of history		Science in general	
**Dependent variables**	EMM	SE	EMM	SE	EMM	SE	EMM	SE	EMM	SE
Scientific norms overall	5.46	.05	5.42	.05	5.21	.05	5.25	.05	5.32	.05
Communalism	5.52	.07	5.58	.07	5.17	.07	5.28	.07	5.18	.07
Disinterestedness	5.31	.06	5.30	.06	5.18	.06	5.15	.06	5.20	.06
Organized skepticism	5.56	.06	5.43	.06	5.36	.06	5.36	.06	5.49	.06
Originality	5.50	.07	5.38	.07	5.09	.08	5.11	.08	5.37	.07

*N* = 1007.

In summary, endorsement of scientific norms seems to depend on the science field, with climate change and virology having higher endorsement than astrophysics and the science of history. For norms overall and most norm subscales, we found significant differences between either virology with a focus on COVID-19 or climate science with science of history and/or astrophysics. That means that scientists from controversial disciplines are especially expected to comply with the norm of communalism (i.e. making results accessible and being transparent regarding research data), organized skepticism (i.e. being critical concerning new evidence potentially changing their conclusions, incurring (scientific) censure on own works), and the norm of originality (i.e. conducting research that is inventive and (practically and/or theoretically) relevant). Regarding comparisons between controversial research areas and science in general, we found significant differences for the norm of communalism. Both virology with a focus on COVID-19 and climate science are confronted with higher support. We think that this norm is seen as inevitable not necessarily for science in general, but for disciplines having immediate impact on society if as much research as possible is available to relevant stakeholders and the public. Interestingly, we did not find any differences regarding the norm of disinterestedness. A possible explanation could be that missing egoistic motivations and general objectivity are seen as fundamental preconditions for all kinds of scientific disciplines and scientists.

### Testing the hypotheses and the research questions

Before analyzing the hypotheses and the research questions, we collapsed the two conditions for uncontroversial fields (combining astrophysics and science of history) and controversial fields (combining virology with a focus on COVID-19 and climate science), leaving us with *n* = 798 cases for the following analyses. In five separate regressions, we then entered age, sex, education, interest in science, experience with science (RQ1a), political ideology (H2), science news consumption across media types (RQ1b), and science field (H1) as predictors for scientific norms overall (mean index of 21 items) and the four subscales (communalism, disinterestedness, organized skepticism, originality). To test interactions, we centered political ideology and science news consumption across media types and computed interaction terms with the science field. Table 3 gives an overview of Step 1 and 2 of the regression analyses.

**Table 3.** Ordinary least squares regression of age, sex, education, interest in science, experience with science, political ideology, science news consumption, field of science (controversial or uncontroversial) predicting public endorsement of scientific norms.

	Scientific norms overall				Communalism				Disinterestedness				Organized skepticism				Originality			
	*b*	*SE*	β	*p*	*b*	*SE*	β	p	b	SE	β	p	b	SE	β	p	b	SE	β	*p*
**Step 1**																				
Constant	3.48	.12		<.001	3.45	.16		<.001	3.31	.14		<.001	3.56	.14		<.001	3.67	.17		<.001
Age	.01	.001	.21	<.001	.01	.002	.20	<.001	.01	.002	.20	<.001	.01	.002	.17	<.001	.01	.002	.11	.002
Sex (1 = female)	.15	.05	.08	.005	.08	.07	.04	.272	.13	.06	.07	.033	.15	.06	.08	.014	.27	.08	.12	<.001
Education (1 = higher)	.20	.05	.11	<.001	.10	.08	.04	.195	.34	.07	.16	<.001	.28	.06	.14	<.001	−.10	.08	−.05	.206
Interest in science	.23	.02	.40	<.001	.23	.03	.33	<.001	.22	.02	.33	<.001	.23	.02	.35	<.001	.212	.03	.30	<.001
Experience with science	.04	.06	.02	.537	.03	.09	.01	.745	.08	.08	.03	.297	.10	.08	.04	.166	−.17	.10	−.07	.071
Political ideology (1 = right-leaning)	−.04	.02	−.08	.009	−.04	.02	−.05	.108	−.05	.02	−.07	.021	−.06	.02	−.10	.001	−.01	.03	−.01	.747
Science news consumption	−.20	.02	−.29	<.001	−.17	.03	−.20	<.001	−.26	.03	−.32	<.001	−.25	.03	−.32	<.001	−.03	.034	−.04	.321
Field of science (1 = controversial)	.21	.05	.12	<.001	.32	.07	.14	<.001	.14	.06	.07	.020	.15	.06	.08	.011	.32	.08	.14	<.001
Ideology × field of science	.03	.05	.04	.370	−.01	.05	−.01	.812	.07	.04	−.08	.070	.08	.04	−.09	.040	−.10	.05	−.10	.036
Science news × field of science	−.02	.04	−.02	.643	−.01	.06	−.01	.865	−.01	.05	−.01	.791	−.02	.05	−.02	.615	−.03	.06	−.03	.573
Adjusted *R*^2^	0.33				0.20				0.31				0.30				.111			
*R* ^2^ *change Step 2*	0.001				0.000				0.003				0.004				0.006			
*F*	50.12***				26.82***				44.36***				43.05***				12.96***			
*F* for change in *R*^2^ compared to Model 1	0.48				0.47				1.66				2.17				2.49			

*n* = 798. Unstandardized regression coefficients, standard errors, standardized coefficients, and p value. Sex (0 =male, 1 = female). Education (0 = lower, 1 = higher). Experience with science (0 = no experience, 1 = experience). Political ideology (0 = left-leaning, 1 = right-leaning). Field of science (0 = uncontroversial, 1 = controversial).

*** p < .001.

First, sociodemographic characteristics predicted the endorsement of scientific norms. Increasing age was a positive predictor for the endorsement of scientific norms overall (*b* = .01, *SE* = .001, *ß* = .21, *p* < .001) and all sub-dimensions (communalism: *b* = .01, *SE* = .002, *ß* = .20, *p* < .001; disinterestedness: *b* = .01, *SE* = .002, *ß* = .20, *p* < .001; organized skepticism: *b* = .01, *SE* = .002, *ß* = .17, *p* < .001; originality: *b* = .01, *SE* = .002, *ß* = .11, *p* = .002). Sex also was an important predictor as females showed significantly more endorsement of scientific norms (*b* = .15, *SE* = .05, *ß* = .08, *p* < .001) and all subdimensions besides communalism (see Table 3; disinterestedness: *b* = .13, *SE* = .06, *ß* = .07, *p* = .033; organized skepticism: *b* = .15, *SE* = .06, *ß* = .08, *p* = .014; originality: *b* = .27, *SE* = .08, *ß* = .12, *p* < .001). Higher education was a positive predictor for the endorsement of scientific norms overall (*b* = .20, *SE* = .05, *ß* = .11, *p* < .001), disinterestedness (*b* = .34, *SE* = .07, *ß* = .16, *p* < .001), and organized skepticism (*b* = .28, *SE* = .06, *ß* = .14, *p* < .001), but not for communalism and originality (see Table 3). Interest in science was a positive predictor across all scales (scientific norms overall: *b* = .23, *SE* = .02, *ß* = .40, *p* < .001; communalism: *b* = .23, *SE* = .03, *ß* = .33, *p* < .001; disinterestedness: *b* = .22, *SE* = .02, *ß* = .33, *p* < .001; organized skepticism: *b* = .23, *SE* = .02, *ß* = .35, *p* < .001; originality: *b* = .21, *SE* = .03, *ß* = .30, *p* < .001). In contrast, personal experience with science was not a significant predictor for any of the scales (see Table 3) (RQ1a). Political ideology acted as significant negative predictor, meaning that people with more liberal than conservative views tended to show more endorsement of scientific norms overall (*b* = −.04, *SE* = .02, *ß* = −.08, *p* = 009), disinterestedness (*b* = −.05, *SE* = .02, *ß* = −.07, *p* = .001), and organized skepticism (*b* = −.01, *SE* = .03, *ß* = −.01, *p* = .001), but not regarding communalism and originality (see Table 3). This supports H2, albeit with some limitations.

Science news consumption across media types appeared to be a negative predictor of the endorsement of scientific norms (RQ1b). People with less consumption of science news show more endorsement of scientific overall (*b* = –.20, *SE* = .02, *ß* = –.29, *p* < .001), communalism (*b* = –.17, *SE* = .03, *ß* = –.20, *p* < .001), disinterestedness (*b* = –.26, *SE* = .03, *ß* = –.32, *p* < .001), and organized skepticism (*b* = –.25, *SE* = .03, *ß* = –.32, *p* < .001), but not for originality (see Table 3). This is a quite surprising result, as science news consumption across media types shows high positive correlations with other positive predictors for the endorsement of scientific norms (e.g. education, interest in science, see Table 1). We therefore additionally calculated correlations between the 10 individual media items and the endorsement of scientific overall: newspaper: *r* = –.03, n.s.; magazines: *r* = –.13, *p* < .001; radio: *r* = –.19, *p* < .001; TV: *r* = –.11, *p* < .001; social media: *r* = –.23, *p* < .001; Wikipedia: *r* = –.13, *p* < .001; Internet sites on scientific topics: *r* = –.10, *p* = .00; podcasts: *r* = –.24, *p* < .001; messaging services: *r* = –.32, *p* < .001; scientific publications: *r* = –.19, *p* < .001. This suggests that the negative association we found clearly varies by medium, with the strongest correlations showing with the consumption of “newer” and probably less professional science communication media like social media, podcasts, and messaging services.

The science field significantly influences scientific norms overall (*b* = .21, *SE* = .05, *ß* = .12, *p* < .001) and all subdimensions (communalism: *b* = .32, *SE* = .07, *ß* = .14, *p* < .001; disinterestedness: *b* = .14, *SE* = .06, *ß* = .07, *p* = .020; organized skepticism: *b* = .15, *SE* = .06, *ß* = .08, *p* = .011; originality: *b* = .32, *SE* = .08, *ß* = .12, *p* < .001). This supports H1 and underlines the trends we found on level of single disciplines, indicating that for controversial fields, the endorsement of scientific norms is generally higher.

**Figure 1.**
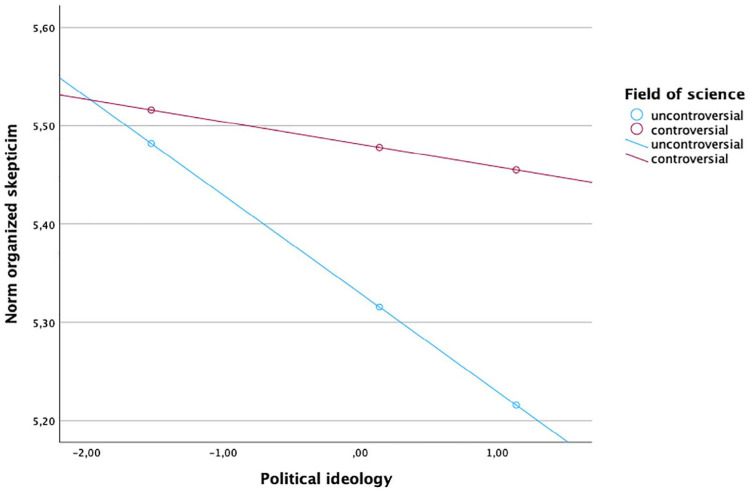
Interaction of organized skepticism, political ideology, and field of science. Note: For color figure please see online version.

**Figure 2.**
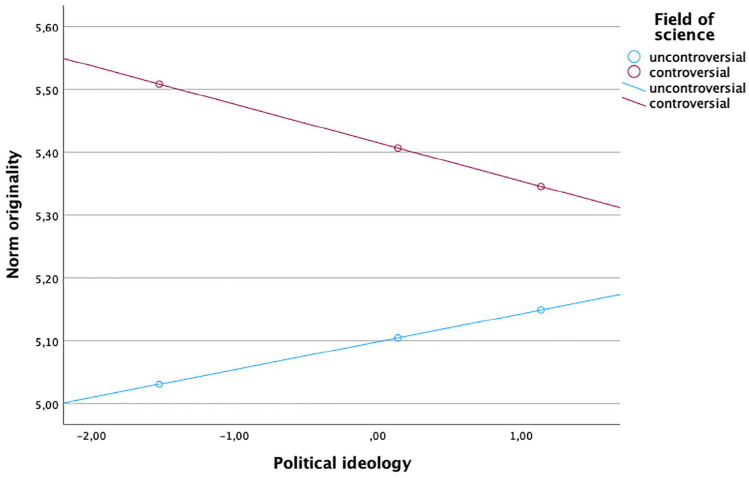
Interaction of originality, political ideology, and field of science. Note: For color figure please see online version.

To examine RQ2 and RQ3, we included interactions in Step 2 of the regressions. The interaction between science field and science news consumption across media types was not related to the overall norm scale or its subdimensions (see Table 3). The interaction between science field and ideology was statistically significant only in two subdimensions: organized skepticism (*b* = .08, *SE* = .04, *p* = .04) and originality (*b* = –.10, *SE* = .05, *p* = .036). Examining the interaction, we can see in Figure 1 that the relationship between more right-leaning ideology is associated with lower levels of organized skepticism in controversial areas compared to uncontroversial areas. For originality, we see a different effect: For controversial fields, scores are lower for right-leaning ideology, while for uncontroversial fields, they have a slight tendency to increase with right-leaning ideology (see Figure 2).

In summary, there are significant tendencies that endorsement of scientific norms is higher for older people, females, people with higher education, people with greater interest in science, people with more liberal worldviews, and people that consume less science news—the latter deriving the strongest from people’s consumption of newer and probably less professional science communication media like social media, or messaging services (see above). Science field also turned out to be a predictor of the endorsement of scientific norms as the norms were more supported for controversial areas. However, to precisely classify these effects, differences between individual norms need to be considered—for instance, sex mattered for the endorsement of all norms besides communalism (see above).

## 5. Discussion

We argue that the endorsement of scientific norms functions as a relevant outcome of science communication that is intertwined with the public understanding and image of science (Johnson and Dieckmann, 2020). Considering scientific norms as important cues for the public understanding of science, their endorsement also implies a basic understanding of how scientific processes work and may also be a positive predictor for scientific literacy. Empirically, we found that the science field is a highly significant predictor for the endorsement of scientific norms, with higher endorsement of scientific norms in controversial areas. We believe that this stems from the important societal function of COVID-19 or climate change research, in which scientists are held to a higher standard to conduct normatively “sound” research. Scientists in these research areas are expected to provide science-based solutions to societally important questions—especially since this science has far-reaching implications for our standard of living and its results are used to justify policies that restrict personal freedom. As a result, scientists in controversial areas are under great pressure to produce reliable knowledge that is held to a higher standard than in less controversial areas. While this is plausible and justified, it can lead to standards that are impossible to meet—and tarnish any scientist, no matter how ethical, in the eyes of the public (Mercer, 2018). In this case, public debate about norms can easily be turned against science and scientists by constructing and inflating norm violations (Cloud, 2020).

Besides the science field, we additionally tested and found several other relevant predictors of the endorsement of scientific norms, including sociodemographic characteristics, interest in science, and science news consumption. For further research in this area, we recommend including those predictors when researching scientific norms and their support by broader publics. This is also true for the effects of political ideology we found. Our results are in line with Lewandowsky and Oberauer (2021) and point to the conclusion that the endorsement of scientific norms may rather reflect political beliefs related to science than concrete expectations for normative behavior, with high endorsement representing positive beliefs about science and low endorsement representing the rejection of established scientific endeavor. In any case, previous research has shown that conservative and right-wing worldviews also predict populistic attitudes, especially those that are related to science (Eberl et al., 2023). Accordingly, further research may consider integrating the public endorsement of scientific norms into the concept of science-related populism (e.g. Bellolio, 2024) and investigate relationships between both constructs.

However, we also want to mention limitations of our study. Besides general critique regarding the foundation of research on scientific norms based on Merton’s framework (see section “Scientific Norms”), this concerns our survey design and measures.

Regarding the ideology measure, we saw that we had a very slight skew to left-leaning ideology (*M* = 4.88, *SD* = 1.57, see Table 1). To make sure that ideology was not unevenly distributed across the experimental conditions, we conducted an analysis of variance (ANOVA; *F* (4, 1002) = .936, *p* = .442), indicating that the slight skew did not affect one condition specifically, but was present in all conditions.

Moreover, we included only four examples for science fields that may be very specific and may not perfectly represent “controversial” and “uncontroversial” research areas (Drummond and Fischhoff, 2020; Erviti et al., 2018). The effects we found therefore need to be tested with more examples of controversial and uncontroversial disciplines. In addition, our survey targeted the German population that is comparable to other countries only to a limited extent due to specific historical and cultural developments in the context of science communication (Peters et al., 2020). Regarding our measures for science news consumption, the instruction for respondents asked whether science content was encountered in diverse media (see Supplemental Material Appendix I). Doing so, we did not measure direct exposure to science. Also, what participants considered to be “science content” was open to interpretation. Self-report often suffers from some imprecision, be it due to labels used to describe the content, social desirability, or inaccurate memory. The category of “science content” may have been especially problematic, since what science is and is not, may be even less clear to respondents than categories such as politics or economy—a fundamental confusion that may be exacerbated by fake science and pseudo-science. Moreover, when building the science news consumption scale, we combined very different sources, which resulted in relatively low mean scores. We ultimately treated the scale as pseudo-metric for correlation and regression analysis. This approach, even though it may be quite common, is open for debate (see Rhemtulla et al., 2012; Robitzsch, 2020). Furthermore, regarding our measures for the endorsement of scientific norms, we found high mean values across all scales. This is not untypical of self-reports about norms—after all, it seems common sense to demand that scientists should be transparent, fair, critical, and have clear motives and progressive research ideas, especially since the barriers for these norms are not salient to non-scientists.

In addition, in our operationalization of originality, we mainly interpreted the concept as relating to theoretical and practical relevance and progress while neglecting to address novelty of the research more directly. This may also contribute to explaining the lower originality endorsement values we found for less controversial fields with less direct relevance to the daily life of most people (see Section “Findings”).

Also, our subscale for universalism could not be created with sufficient reliability. In consequence, our overall mean index for scientific norms does not represent the full scientific ethos (Merton, 1973). Possibly, the items we combined from three different sources (see Supplemental Material Appendix “Scales and items”: Anderson, 2000; Bray and Von Storch, 2017; Lewandowsky and Oberauer, 2021) did not represent one dimension but several: All items refer to the scientific need to refrain from potential biases to ensure high-quality scientific data. However, the items as such reference different biases (discrimination, personal sentiment, and reputation) that were answered in a different way, implicating that respondents possibly weight the importance of differed biases against each other. Nonetheless, the idea of the universalism dimension seems important and a new instrument to capture it is needed for future research. In any case, we find it essential to differentiate between single norms as we found differences when comparing their respective effects. Finally, the distinction between disciplines enabled rich insight into how the public views and evaluates scientific work. Thus, we find it useful to include differentiations between different scientific disciplines in research on the public image and understanding of science, especially those that are more controversial and visible within society having a more direct influence on people’s lives and those that appear to be less controversial in this regard.

## 6. Conclusion

Merton’s norms have aged with grace, but they have aged. Calls for re-formulating and transforming them to fit modern science better and reflect disciplinary differences are being discussed, for example, regarding the implications of open science practices (Hosseini et al., 2024), the meaning of objectivity (Freese and Peterson, 2018), and the interplay of social media and science communication as an ambivalent enterprise (Hogan and Sweeney, 2013) as well as the dissociation of certification in peer-review and amplification through digital technologies of sharing content independently of scientific journals (Lee, 2022). Kønig et al. (2017) invoke the inception of an “ethos of post-normal science”—norms and values regarding science advice practices in situations where an effective management of scientific uncertainty is crucial for solving practical problems. Kønig et al. (2017) differentiate such practices of post-normal science from “normal” science aiming at certain knowledge, not decision-making. However, post-normal science does not contradict but complements normal science (Kønig et al., 2017). Our study has shown that, indeed, societal problems such as COVID-19 or climate change may provoke situations in which post-normal norms of science might be needed to guide scientists’ actions as the public evaluates such scientists involved in problem-solving against higher normative standards.

## Supplemental Material

sj-docx-1-pus-10.1177_09636625251315882 – Supplemental material for Endorsement of scientific norms among non-scientists: The role of science news consumption, political ideology, and science field Supplemental material, sj-docx-1-pus-10.1177_09636625251315882 for Endorsement of scientific norms among non-scientists: The role of science news consumption, political ideology, and science field by Markus Schug, Helena Bilandzic and Susanne Kinnebrock in Public Understanding of Science

## Notes

1. This analysis is part of a larger project. A different portion of the data focusing on public perceptions of trustworthiness and authenticity toward scientists in controversial scientific fields is presented elsewhere (Schug et al., 2024).
