# Supplementary material for: Endorsement of scientific norms among non-scientists: The role of science news consumption, political ideology, and science field
Source: Public Underst Sci. 2025 Feb 28;34(6):752–69. doi: 10.1177/09636625251315882 (PMC12274562; doi:10.1177/09636625251315882)
Supplement: sj-docx-1-pus-10.1177_09636625251315882 – Supplemental material for Endorsement of scientific norms among non-scientists: The role of science news consumption, political ideology, and science field [file sj-docx-1-pus-10.1177_09636625251315882.docx]

**Endorsement of scientific norms among non-scientists**

**The role of science news consumption, political ideology, and science field**

(Supplemental material)

Markus Schug, Helena Bilandzic, and Susanne Kinnebrock

Appendix I: Scales and items

# Appendix I: Scales and items

**Interest in science (**Marschall et al., 2011; Retzbach & Maier, 2015^[[1]](#footnote-1)^)

Instruction: Nun würden wir gerne wissen, inwiefern Sie an Wissenschaft und Forschung interessiert sind. Wie würden Sie die folgenden Aussagen einstufen? [Now we would like to know to what extent you are interested in science and research. How would you rate the following statements?]

| **Item** | **Translation** |
| --- | --- |
| Ganz allgemein gesprochen interessiere ich mich sehr für Wissenschaft. | Generally speaking, I am very interested in science. |
| Für mich ist die Wissenschaft ein spannendes Thema. | For me, science is an exciting subject. |
| Neue wissenschaftliche Entdeckungen und Forschungen verfolge ich mit großer Neugier. | I pursue new scientific discoveries and research with great curiosity. |
| Medien- und Internetbeiträge über Wissenschaft und Forschung interessieren mich sehr. | Media and internet articles about science and research interest me a lot. |

Note. 7-point agreement scales from 1 = “do not agree at all” to 7 = “totally agree”

**Experiences with science** (Wissenschaft im Dialog, 2021)

Instruction: Nun möchten wir noch etwas über Ihren persönlichen Bezug zu Wissenschaft und Forschung erfahren [Now we would like to know something about your personal relationship to science and research].

Kennen Sie eine Wissenschaftlerin oder einen Wissenschaftler persönlich? [Do you know a scientist personally?]

| □ | nein [no] |
| --- | --- |
| □ | ja [yes] |

Arbeiten Sie selbst in Wissenschaft und Forschung [Do you work in science and research yourself?]

| □ | nein, ich habe noch nie in Wissenschaft und Forschung gearbeitet [no, I have never worked in science and research before] |
| --- | --- |
| □ | nein, aber ich habe in der Vergangenheit in Wissenschaft und Forschung gearbeitet [no, but I worked in science in the past] |
| □ | ja [yes] |

**Political ideology** (Huber et al., 2019)

Instruction: Viele Leute verwenden die Begriffe ‚links‘ und ‚rechts‘, wenn um unterschiedliche politische Einstellungen geht. Wie würden Sie Ihre Sicht auf politische, soziale und wirtschaftliche Angelegenheiten beschreiben? [Many people use the terms ‘left’ and ‘right’ to characterize different political attitudes. How would you describe your views on political, economic, and social issues?]

| **Item/Question** | **Translation** |
| --- | --- |
| Wo würden Sie sich bei *politischen* Angelegenheiten auf einer Skala von 1-10 einordnen (1 = ‚links‘ und 10 = ‚rechts‘)? | Where would you rank yourself on *political* issues on a scale of 1-10 (1 = 'left' and 10 = 'right')? |
| Wo würden Sie sich bei *sozialen* Angelegenheiten auf einer Skala von 1-10 einordnen (1 = ‚links‘ und 10 = ‚rechts‘)? | Where would you rank yourself on *economic* issues on a scale of 1-10 (1 = 'left' and 10 = 'right')? |
| Wo würden Sie sich bei *wirtschaftlichen* Angelegenheiten auf einer Skala von 1-10 einordnen (1 = ‚links‘ und 10 = ‚rechts‘)? | Where would you rank yourself on *social* issues on a scale of 1-10 (1 = 'left' and 10 = 'right')? |

Note. 10-point scales from 1 = 'left' to 10 = 'right'

**Science news consumption**

Instruction: In den Medien wird ja über viele wissenschaftliche Themen berichtet. Wie oft sind Sie in den letzten 2 Wochen in den folgenden Medien auf Informationen oder andere Inhalte aus der Wissenschaft gestoßen? [Media report a lot about scientific topics. In the past two weeks, how often did you encounter information or contents from science in the following media?]

| **Item** | **Translation** |
| --- | --- |
| Zeitung (Print, Online, mobile App) | Newspaper (print, online, mobile app) |
| Zeitschriften (Print, Online, mobile App) | Magazines (print, online, mobile app) |
| Radio (inklusive Streaming, Online, mobile App) | Radio (including Streaming, online, mobile app) |
| Fernsehen (inklusive Streaming, Online, mobile App) | TV (including Streaming, online, mobile app) |
| Soziale Medien (z. B. Facebook, Twitter, Instagram) | Social media (e.g., Facebook, Twitter, Instagram) |
| Wikipedia | Wikipedia |
| Internetseiten über wissenschaftliche Themen | Internet sites on scientific topics |
| Podcasts | Podcasts |
| Nachrichtenübermittlungsdienste (z. B. WhatsApp, SMS) | Messaging services (e.g., WhatsApp, SMS) |
| Wissenschaftliche Veröffentlichungen | Scientific publications |

Note. 8-point scales (“not at all” (1), “a few times” (2), “about once a week” (3), “a few times per week” (4), “nearly every day” (5), “every day” (6), “a few times per day” (7) to “many times per day” (8))

**Endorsement of scientific norms (**Adapted from Anderson, 2000; Bray & von Storch, 2017; Lewandowsky & Oberauer, 2021; Merton, 1973; Ziman, 2000)

Instruction: Im Folgenden finden Sie eine Liste von Aussagen zu Strukturen und Arbeitsweisen in der *virologischen Forschung zu COVID-19* / *Klimaforschung* / *Astrophysik* / *Geschichtswissenschaft* / *Wissenschaft*. Diese spiegeln unterschiedliche Vorstellungen wider, wie die *virologische Forschung zu COVID-19* / *Klimaforschung* / *Astrophysik* / *Geschichtswissenschaft* / *Wissenschaft* funktionieren sollten. Bitte entscheiden Sie für jede Aussage einzeln, inwiefern Sie dieser zustimmen. Wenn Sie denken, dass eine Aussage voll und ganz zutrifft, vergeben Sie eine 7. Wenn Sie denken, dass eine Aussage ganz und gar nicht zutrifft, vergeben Sie eine 1. Mit den Zahlen dazwischen können Sie Ihre Meinung abstufen. [The following is a list of statements about structures and ways of working in *virology with a focus on COVID-19* / *climate science* / *astrophysics* / *science of history* / *science*. These reflect different ideas about how *virology with a focus on COVID-19* / *climate science* / *astrophysics* / *science of history* / *science* should work. Please decide for each statement individually to what extent you agree with it. If you think a statement is completely true, assign a 7. If you think a statement is completely not true, assign a 1. You can use the numbers in between to grade your opinion].

| **Communalism** | | | |
| --- | --- | --- | --- |
| **Adapted items** | **Original item(s)** | **Source and original scale** | **Notes** |
| Erkenntnisse aus der *virologischen Forschung zu COVID-19* / *Klimaforschung* / *Astrophysik* / *Geschichtswissenschaft* / *Wissenschaft* sollten allen Menschen weltweit zur Verfügung stehen.  [Findings from *virology with a focus on COVID-19* / *climate science* / *astrophysics* / *science of history* / *science* should be available to everybody everywhere in the world.] | Scientific findings should be available to everybody everywhere in the world. | Lewandowsky & Oberauer (2021)  7-point scale (1 = “strongly disagree”; 7 = “strongly agree”) |  |
| Ergebnisse aus der *virologischen Forschung zu COVID-19* / *Klimaforschung* / *Astrophysik* / *Geschichtswissenschaft* / *Wissenschaft* sollten öffentliches Eigentum sein.  [Results from *virology with a focus on COVID-19* / *climate science* / *astrophysics* / *science of history* / *science* should be public property.] | Scientific results should be… | Bray & von Storch (2017)  7-point scale (1 = “private”; 7 = “public property”) |  |
| Es ist völlig inakzeptabel, wenn *Virologinnen und Virologen, die speziell zu COVID-19 forschen,* / *Klimaforscherinnen und -forscher* / *Astrophysikerinnen und Astrophysiker* / *Geschichtswissenschaftlerinnen und -wissenschaftler / Wissenschaftlerinnen und Wissenschaftler* Informationen verbergen, die für andere wissenschaftlich Tätige von Bedeutung sein könnten.  [It is completely unacceptable for *virologists researching COVID-19* / *climate researchers* / *astrophysicists* / *historians* / *scientists* to hide information might be of importance to other scientific workers.] | To hide information which might be of importance to other scientists is… | Bray & von Storch (2017)  7-point scale (1 = “totally acceptable”;7 = “completely unacceptable”) |  |
| *Virologinnen und Virologen, die speziell zu COVID-19 forschen,* / *Klimaforscherinnen und -forscher* / *Astrophysikerinnen und Astrophysiker* / *Geschichtswissenschaftlerinnen und -wissenschaftler / Wissenschaftlerinnen und Wissenschaftler* sollten die Eigentumsrechte an ihren Forschungsergebnissen um jeden Preis schützen.  [*Virologists researching COVID-19* / *climate researchers* / *astrophysicists* / *historians* / *scientists* should protect the property rights to their research results at all costs.] | It is important to protect my individual scientific property rights. | Bray & von Storch (2017)  7-point scale 1 = “not at all”  7 = “at all costs” | Reverse coded  item eliminated because of a lack of consistency with the construct |
| *Virologinnen und Virologen, die speziell zu COVID-19 forschen,* / *Klimaforscherinnen und -forscher* / *Astrophysikerinnen und Astrophysiker* / *Geschichtswissenschaftlerinnen und -wissenschaftler / Wissenschaftlerinnen und Wissenschaftler* sollten ihre Forschungsdaten und -materialien unverzüglich und ohne Einschränkungen offenlegen, selbst wenn sie dadurch Gefahr laufen, dass ihre Ideen gestohlen werden.  [*Virologists researching COVID-19* / *climate researchers* / *astrophysicists* / *historians* / *scientists* should disclose their research data and materials immediately and without restriction, even if it means risking their ideas being stolen.] | Other scientists should have free access to my data after I have published the initial findings. | Bray & von Storch (2017)  7-point scale (1 = “absolutely not”; 7 = “under all circumstances”) |  |
|  | I have the right to keep initial findings secret to ensure that I get full credit when the findings are published. | Bray & von Storch (2017)  7-point scale (1 = “absolutely”; 7 = “I have no rights at all”) |  |
| *Virologinnen und Virologen, die speziell zu COVID-19 forschen,* / *Klimaforscherinnen und -forscher* / *Astrophysikerinnen und Astrophysiker* / *Geschichtswissenschaftlerinnen und -wissenschaftler / Wissenschaftlerinnen und Wissenschaftler* sollten neue Erkenntnisse offen mit anderen teilen.  [*Virologists researching COVID-19* / *climate researchers* / *astrophysicists* / *historians* / *scientists* should openly share new findings with colleagues.] | Scientists openly share new findings with colleagues. | Anderson (2000)  3-point scale (“very little or not at all”; “to some extent”; “to a great extent”) on the extent to which it should represent behavior of scientists. |  |

Note. 7-point agreement scales from 1 = “do not agree at all” to 7 = “totally agree”

| **Universalism** | | | |
| --- | --- | --- | --- |
| **Adapted items** | **Original item(s)** | **Source and original scale** | **Notes** |
| Die Wahrheit einer Entdeckung in der *virologischen Forschung zu COVID-19* / *Klimaforschung* / *Astrophysik* / *Geschichtswissenschaft* / *Wissenschaft* sollte unabhängig davon sein, welche Nationalität, welches Geschlecht, welche Ethnie oder welchen Glauben die forschende Person hat.  [The truth of a discovery in *virology with a focus on COVID-19* / *climate science* / *astrophysics* / *science of history* / *science* should be independent of the nationality, gender, ethnicity, or faith of the scientist making it.] | The truth of a scientific discovery does not depend on the nationality, gender, race, or faith of the scientist making it. | Lewandowsky & Oberauer (2021)  7-point scale (1 = “strongly disagree”; 7 = “strongly agree”) | item excluded because universalism-scale was not reliable |
| Die Akzeptanz oder Ablehnung wissenschaftlicher Erkenntnisse und Behauptungen in der *virologischen Forschung zu COVID-19* / *Klimaforschung* / *Astrophysik* / *Geschichtswissenschaft* / *Wissenschaft* sollte von persönlichen Empfindungen abhängen.  [Acceptance or rejection of findings and claims in *virology with a focus on COVID-19* / *climate science* / *astrophysics* / *science of history* / *science* should depend on personal feelings.] | Acceptance or rejection of scientific findings and claims should depend on personal feelings. | Bray & von Storch (2017)  7-point scale (1 = “never”; 7 = “always”) | Reverse coded  item excluded because universalism-scale was not reliable |
| *Virologinnen und Virologen, die speziell zu COVID-19 forschen,* / *Klimaforscherinnen und -forscher* / *Astrophysikerinnen und Astrophysiker* / *Geschichtswissenschaftlerinnen und -wissenschaftler / Wissenschaftlerinnen und Wissenschaftler* sollten Forschung nur nach ihrem Wert, d. h. nach anerkannten Standards des Fachgebiets, bewerten.  [*Virologists researching COVID-19* / *climate researchers* / *astrophysicists* / *historians* / *scientists* should evaluate research only on its merits, i.e., according to accepted standards of the field.] | Scientists evaluate research only on its merit, i.e., according to accepted standards of the field. | Anderson (2000)  3-point scale (“very little or not at all”; “to some extent”; “to a great extent”) on the extent to which it should represent behavior of scientists. | item excluded because universalism-scale was not reliable |
| *Virologinnen und Virologen, die speziell zu COVID-19 forschen,* / *Klimaforscherinnen und -forscher* / *Astrophysikerinnen und Astrophysiker* / *Geschichtswissenschaftlerinnen und -wissenschaftler / Wissenschaftlerinnen und Wissenschaftler* sollten neues Wissen und seine Einsatzgebiete auf der Grundlage des Rufs und der bisherigen Aktivitäten der durchführenden Forscherinnen und Forscher bewerten.  [*Virologists researching COVID-19* / *climate researchers* / *astrophysicists* / *historians* / *scientists* should assess new knowledge and its applications based on the reputation and past productivity of the conducting researchers.] | Scientists assess new knowledge and its applications based on the reputation and past productivity of the individual or research group. | Anderson (2000)  3-point scale (“very little or not at all”; “to some extent”; “to a great extent”) on the extent to which it should represent behavior of scientists. | Reverse coded  item excluded because universalism-scale was not reliable |

Note. 7-point agreement scales from 1 = “do not agree at all” to 7 = “totally agree”

| **Disinterestedness** | | | |
| --- | --- | --- | --- |
| **Adapted items** | **Original item(s)** | **Source and original scale** | **Notes** |
| *Virologinnen und Virologen, die speziell zu COVID-19 forschen,* / *Klimaforscherinnen und -forscher* / *Astrophysikerinnen und Astrophysiker* / *Geschichtswissenschaftlerinnen und -wissenschaftler / Wissenschaftlerinnen und Wissenschaftler* sollten Beweise über ihre eigenen Ansichten stellen.  [*Virologists researching COVID-19* / *climate researchers* / *astrophysicists* / *historians* / *scientists* should put evidence ahead of their own views.] | Scientists should put evidence ahead of their own views. | Lewandowsky & Oberauer (2021)  7-point scale (1 = “strongly disagree”; 7 = “strongly agree”) |  |
| *Virologinnen und Virologen, die speziell zu COVID-19 forschen,* / *Klimaforscherinnen und -forscher* / *Astrophysikerinnen und Astrophysiker* / *Geschichtswissenschaftlerinnen und -wissenschaftler / Wissenschaftlerinnen und Wissenschaftler* sollten Forschung betreiben, die ausschließlich ihren persönlichen Interessen folgt.  [*Virologists researching COVID-19* / *climate researchers* / *astrophysicists* / *historians* / *scientists* should conduct research that is only of personal interest to them.] | I pursue research that is only of personal interest to me. | Bray & von Storch (2017)  7-point scale (1 = “never”; 7 = “always”) | Reverse coded |
| *Virologinnen und Virologen, die speziell zu COVID-19 forschen,* / *Klimaforscherinnen und -forscher* / *Astrophysikerinnen und Astrophysiker* / *Geschichtswissenschaftlerinnen und -wissenschaftler / Wissenschaftlerinnen und Wissenschaftler* sollten versuchen sicherzustellen, dass ihre intellektuelle Arbeit unabhängig ist von persönlichen Überzeugungen und Werten.  [*Virologists researching COVID-19* / *climate researchers* / *astrophysicists* / *historians* / *scientists* should try to ensure that their intellectual work is not influenced by their personal beliefs and values.] | I try to ensure that my intellectual work is not influenced by my personal beliefs and values. | Bray & von Storch (2017)  7-point scale (1 = “never”; 7 = “always” |  |
| *Virologinnen und Virologen, die speziell zu COVID-19 forschen,* / *Klimaforscherinnen und -forscher* / *Astrophysikerinnen und Astrophysiker* / *Geschichtswissenschaftlerinnen und -wissenschaftler / Wissenschaftlerinnen und Wissenschaftler* sollten ihre Forschungsinteressen den verfügbaren Fördergeldern anpassen.  [*Virologists researching COVID-19* / *climate researchers* / *astrophysicists* / *historians* / *scientists* should align their research interests with available funding opportunities.] | How often do you have no choice but to align your research interests with funding opportunities. | Bray & von Storch (2017)  7-point scale (1 = “never”; 7 = “always”) | Reverse coded |
| *Virologinnen und Virologen, die speziell zu COVID-19 forschen,* / *Klimaforscherinnen und -forscher* / *Astrophysikerinnen und Astrophysiker* / *Geschichtswissenschaftlerinnen und -wissenschaftler / Wissenschaftlerinnen und Wissenschaftler* sollten durch den Wunsch nach Wissen und Entdeckung motiviert sein.  [*Virologists researching COVID-19* / *climate researchers* / *astrophysicists* / *historians* / *scientists* should be motivated by a desire for knowledge and discovery.] | Scientists are motivated by the desire for knowledge and discovery, and not by the possibility of personal gain. | Anderson (2000)  3-point scale (“very little or not at all”; “to some extent”; “to a great extent”) on the extent to which it should represent behavior of scientists. |  |
| *Virologinnen und Virologen, die speziell zu COVID-19 forschen,* / *Klimaforscherinnen und -forscher* / *Astrophysikerinnen und Astrophysiker* / *Geschichtswissenschaftlerinnen und -wissenschaftler / Wissenschaftlerinnen und Wissenschaftler* sollten durch möglichen persönlichen Nutzen (finanzielle, politische oder karrierebezogene Ziele) motiviert sein.  [*Virologists researching COVID-19* / *climate researchers* / *astrophysicists* / *historians* / *scientists* should be motivated by potential personal benefit (financial, political, or career-related goals).] |  |  | Reverse coded |

Note. 7-point agreement scales from 1 = “do not agree at all” to 7 = “totally agree”

| **Organized Skepticism** | | | |
| --- | --- | --- | --- |
| **Adapted items** | **Original item(s)** | **Source and original scale** | **Notes** |
| *Virologinnen und Virologen, die speziell zu COVID-19 forschen,* / *Klimaforscherinnen und -forscher* / *Astrophysikerinnen und Astrophysiker* / *Geschichtswissenschaftlerinnen und -wissenschaftler / Wissenschaftlerinnen und Wissenschaftler* sollten immer offenbleiben und bereit sein, ihre eigenen Schlussfolgerungen zu ändern, wenn neue Beweise hinzukommen.  [*Virologists researching COVID-19* / *climate researchers* / *astrophysicists* / *historians* / *scientists* should always keep an open mind and be prepared to change their conclusions if new evidence comes along.] | Scientists should always keep an open mind and be prepared to change their conclusions if new evidence comes along. | Lewandowsky & Oberauer (2021)  7-point scale (1 = “strongly disagree”; 7 = “strongly agree”) |  |
| *Virologinnen und Virologen, die speziell zu COVID-19 forschen,* / *Klimaforscherinnen und -forscher* / *Astrophysikerinnen und Astrophysiker* / *Geschichtswissenschaftlerinnen und -wissenschaftler / Wissenschaftlerinnen und Wissenschaftler* sollten andere Beiträge zu ihrem Forschungsbereich nur nach ihrer Qualität beurteilen.  [*Virologists researching COVID-19* / *climate researchers* / *astrophysicists* / *historians* / *scientists* should judge other contributions to their field of research only on the basis of quality.] | I judge other contributions to my science on the basis of quality only. | Bray & von Storch (2017)  7-point scale (1 = “never”; 7 = “always”) |  |
| *Virologinnen und Virologen, die speziell zu COVID-19 forschen,* / *Klimaforscherinnen und -forscher* / *Astrophysikerinnen und Astrophysiker* / *Geschichtswissenschaftlerinnen und -wissenschaftler / Wissenschaftlerinnen und Wissenschaftler* sollten die Arbeit anderer Forscherinnen und Forscher in erster Linie nach dem Status der Autorin bzw. des Autors bewerten.  [*Virologists researching COVID-19* / *climate researchers* / *astrophysicists* / *historians* / *scientists* should assess the work of other researchers primarily on the basis of the author's status.] | I assess the work of other scientists primarily on the status of the author. | Bray & von Storch (2017)  7-point scale (1 = “never”; 7 = “always”) | Reverse coded |
| *Virologinnen und Virologen, die speziell zu COVID-19 forschen,* / *Klimaforscherinnen und -forscher* / *Astrophysikerinnen und Astrophysiker* / *Geschichtswissenschaftlerinnen und -wissenschaftler / Wissenschaftlerinnen und Wissenschaftler* sollten ihre Forschung an die Erkenntnisse prominenterer Kolleginnen und Kollegen anpassen.  [*Virologists researching COVID-19* / *climate researchers* / *astrophysicists* / *historians* / *scientists* should align their research with the findings of more prominent colleagues.] | How often is there pressure to conform your research to fit with the findings of more prominent scientists? | Bray & von Storch (2017)  7-point scale (1 = “never”; 7 = “always”) | Reverse coded |
| *Virologinnen und Virologen, die speziell zu COVID-19 forschen,* / *Klimaforscherinnen und -forscher* / *Astrophysikerinnen und Astrophysiker* / *Geschichtswissenschaftlerinnen und -wissenschaftler / Wissenschaftlerinnen und Wissenschaftler* sollten alle neuen Beweise, Hypothesen, Theorien und Innovationen berücksichtigen, selbst solche, die ihre eigene Forschung herausfordern oder dieser widersprechen.  [*Virologists researching COVID-19* / *climate researchers* / *astrophysicists* / *historians* / *scientists* should consider all new evidence, hypotheses, theories, and innovations, even those that challenge or contradict their own work.] | Scientists consider all new evidence, hypotheses, theories, and innovations, even those that challenge or contradict their own work. | Anderson (2000)  3-point scale (“very little or not at all”; “to some extent”; “to a great extent”) on the extent to which it should represent behavior of scientists. |  |
| *Virologinnen und Virologen, die speziell zu COVID-19 forschen,* / *Klimaforscherinnen und -forscher* / *Astrophysikerinnen und Astrophysiker* / *Geschichtswissenschaftlerinnen und -wissenschaftler / Wissenschaftlerinnen und Wissenschaftler* sollten dazu bereit sein, ihre Ergebnisse extern prüfen zu lassen.  [V*irologists researching COVID-19* / *climate researchers* / *astrophysicists* / *historians* / *scientists* should be willing to have their results externally reviewed.] | (no item, developed based on basic theory) | Merton (1973); Ziman (2000) |  |
| Ergebnisse aus der *virologischen Forschung zu COVID-19* / *Klimaforschung* / *Astrophysik* / *Geschichtswissenschaft* / *Wissenschaft* sollten stets von unabhängigen Dritten überprüft werden.  Results from *virology with a focus on COVID-19* / *climate science* / *astrophysics* / *science of history* / *science* should always be verified by independent third parties. | (no item, developed based on basic theory) | Merton (1973); Ziman (2000) |  |

Note. 7-point agreement scales from 1 = “do not agree at all” to 7 = “totally agree”

| **Originality** | | | |
| --- | --- | --- | --- |
| **Adapted items** | **Original item(s)** | **Source and original scale** | **Notes** |
| *Virologinnen und Virologen, die speziell zu COVID-19 forschen,* / *Klimaforscherinnen und -forscher* / *Astrophysikerinnen und Astrophysiker* / *Geschichtswissenschaftlerinnen und -wissenschaftler / Wissenschaftlerinnen und Wissenschaftler* sollten Forschung betreiben, die originell ist und einen Fortschritt bringt.  [*Virologists researching COVID-19* / *climate researchers* / *astrophysicists* / *historians* / *scientists* should conduct research that is original and makes a difference.] | (no item, developed based on basic theory) | Ziman (2000) |  |
| *Virologinnen und Virologen, die speziell zu COVID-19 forschen,* / *Klimaforscherinnen und -forscher* / *Astrophysikerinnen und Astrophysiker* / *Geschichtswissenschaftlerinnen und -wissenschaftler / Wissenschaftlerinnen und Wissenschaftler* sollten Forschung betreiben, die von hoher praktischer Relevanz ist.  [*Virologists researching COVID-19* / *climate researchers* / *astrophysicists* / *historians* / *scientists* should conduct research that is of high practical relevance.] | (no item, developed based on basic theory) | Ziman (2000) |  |
| *Virologinnen und Virologen, die speziell zu COVID-19 forschen,* / *Klimaforscherinnen und -forscher* / *Astrophysikerinnen und Astrophysiker* / *Geschichtswissenschaftlerinnen und -wissenschaftler / Wissenschaftlerinnen und Wissenschaftler* sollten Forschung betreiben, die in ihrem Forschungsbereich als relevant gilt.  [*Virologists researching COVID-19* / *climate researchers* / *astrophysicists* / *historians* / *scientists* should conduct research that is considered relevant in their research area.] | (no item, developed based on basic theory) | Ziman (2000) |  |
| *Virologinnen und Virologen, die speziell zu COVID-19 forschen,* / *Klimaforscherinnen und -forscher* / *Astrophysikerinnen und Astrophysiker* / *Geschichtswissenschaftlerinnen und -wissenschaftler / Wissenschaftlerinnen und Wissenschaftler* sollten Methoden und Theorien, die ihnen veraltet erscheinen, verwerfen.  [*Virologists researching COVID-19* / *climate researchers* / *astrophysicists* / *historians* / *scientists* should discard methods and theories that seem outdated to them.] | (no item, developed based on basic theory) | Ziman (2000) | item eliminated because of a lack of consistency with the construct |

Note. 7-point agreement scales from 1 = “do not agree at all” to 7 = “totally agree”


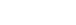


1. The citations refer to the bibliography within the full article. [↑](#footnote-ref-1)
